# Transactions of the Pathological Society of Philadelphia

**Published:** 1839-11-02

**Authors:** C. W. Pennock, E. M. Moore

**Affiliations:** Physician to the Philadelphia Hospital; Philadelphia; Late Resident Physician to the Frankford Asylum; Philadelphia


### MEDICAL EXAMINER.

DEVOTED TO MEDICINE, SURGERY, AND THE COLLATERAL SCIENCES.

No. 44.] PHILADELPHIA, SATURDAY, NOVEMBER 2, 1839. [Vol. II.

### TRANSACTIONS OF THE PATHOLOGICAL SOCIETY OF PHILADELPHIA.

*Report of Experiments upon the Action of the Heart.* By C. W. Pennock, M. D., *Physician to the Philadelphia Hospital, and* E. M. Moore, M. D., *late Resident Physician to the Frankford Asylum.*

*October 28th—November 4th,* 1839.

The President, Dr. Gerhard, in the chair.

Dr. Pennock read the following Report of Experiments upon the Action of the Heart:

Impressed with the importance of the experiments instituted a few years since, to illustrate the heart's action, by some European physiologists, we had resolved, more than a year past, to repeat them upon the first favourable opportunity. We were the more anxious to perform them, as the subject is one that has received but little attention in this country, and the profession seems scarcely aware of its importance. Circumstances, however, prevented us from carrying our designs into effect until a short time since; when upon application to an intelligent victualler in a neighbouring village, every facility was afforded us. We have been assisted by several medical gentlemen; but to Dr. Hardy, of the Philadelphia Hospital, who aided us in all the experiments, may be mainly attributed their successful results. We were also kindly assisted by Dr. Wood, Resident Physician of Frankford Asylum, Dr. Stillé, of Pennsylvania Hospital, and Mr. Burns, of Mobile.

Before proceeding to detail the experiments, we may say that the stethoscopes or ear-trumpets used were flexible, constructed of a coil of wire, covered with gum elastic and silk; one, about four feet long, the ear-piece and hollow cone for the reception of sound, being of horn; the other, about two feet long, the ends composed of block-tin, and smaller than the first. This instrument is essential to the success of the experiment, as the impulse is so great with the ordinary stethoscope, as to render the analysis of sound very uncertain. In measuring the heart, the ordinary shoemaker's measure is used, by which very accurate results may be obtained. Artificial respiration was maintained by the bellows at eighteen to twenty inflations per minute.

*Experiment 1st.*—Present, Drs. Hardy, Wood, Pennock, and Moore.

A ram, about one year old, was selected.

Owing to the alarm of the animal, it was found extremely difficult to ascertain the natural pulse and respiration; but during the time he was most quiet, the former ranged from ninety-six to one hundred and eight per minute, and the latter from thirty to forty in the same time. The stethoscope, applied to the left side of the chest, opposite the fourth rib, revealed the sounds of the heart distinct and normal, but faint. Upon the sternum, in the same line, they could scarcely be distinguished. The animal was then deprived of sensation by several blows upon the anterior portion of the cranium; and the bellows-tube being immediately introduced through an incision in the trachea, respiration was artificially sustained. An incision was then made down upon the sternum, and extending its whole length, with a knife whose edge was purposely roughened to prevent hæmorrhage. The bone was then divided longitudinally by a saw, and its parts separated by hooks, thus presenting a cavity of six or eight inches in diameter. Ten minutes had elapsed from the time the blow was given until the chest was opened, but the heart was still observed to beat irregularly and very rapidly. The excitement, however, soon subsided, and the heart pulsated regularly, and with a frequency of ninety-six per minute. The stethoscope was first applied to the heart—the pericardium being still unopened—and the sounds were observed to be of the same character as previously observed, but much louder. The first sound appeared to occupy about one-half of the whole time of a pulsation; this was followed by the second, which is about one-half as long as the first, or one-fourth of the whole, and is more flapping than the first; the remaining time is occupied by repose.

The head of the auscultator being averted, and his eyes closed, the end of the stethoscope was applied by an assistant to the base near the valves, and to the body of the heart alternately; and it was decided by each in succession, that the first sound was louder over the body of the ventricles than near the valves, while the second sound was much more distinct near the valves, than over the ventricles elsewhere. The change, however, modified the second sound much more than the first. A portion of the lungs being interposed, we found the sounds duller, but in other respects of the same character. The anterior portion of the pericardium was then removed, and the heart exposed, presenting the right ventricle and auricle, and a small portion of the left ventricle, the auricle being concealed behind the heart. During the ventricular systole, the right ventricle was observed to be flattened, and the finger and stethoscope being applied, the first sound and impulse occurred at the same time. During this contraction the base of the heart revolved for a short distance to the left, supposed to be about one-sixteenth of a circle, while the apex turned to the right at the same moment, thus causing the heart to assume a spiral form during the systole. The transverse diameter was much diminished by this systole; during diastole it increased and assumed a rounded appearance.

The stethoscope was again applied in the same manner as heretofore detailed, and with the same result. A comparison being instituted, with the head averted as before, between the character of the sounds over the right and left ventricle, it was unanimously conceded, that on the right, the first sound was flapping and shorter than on the left, while on the latter it was prolonged and rushing. Such was the rapidity of the heart's action, that some difference of opinion exists with reference to the relative contraction of the auricle and ventricle. Drs. Pennock and Wood being of the opinion that the ventricular systole is immediately followed by the auricular contraction, which is synchronous with the ventricular diastole; or to detail the succession more accurately, we have, 1st, the systole of the ventricles occupying one-half of the whole time, during which systole the auricle dilates; 2d, immediately at the termination of the systole, the auricle contracts, and the ventricle dilates, synchronously, occupying one-fourth of the whole time; 3d, the state of repose follows, in which the ventricle is full, occupying the remaining time. Dr. Pennock is of the opinion that the auricular contraction occupied rather less time than the period of repose. Dr. Moore coincides with this opinion, except in not considering the emptying of the auricles, during the diastole of the ventricles, the result of active auricular contraction, but of simple distention, relieved by the diastole of the ventricle, and thinks he perceives a contraction of the auricle at the termination of repose, immediately preceding the ventricular systole. The first sound, impulse, and ventricular systole, were synchronous. There was, however, an appreciable difference between the contraction of the ventricles and the pulse, increasing as the distance from the heart was greater. The pulse varied from eighty-four to ninety-six, becoming irregular when the artificial respiration was omitted or too rapid.

The heart pulsated two hours after opening the chest.

*Experiment 2d.*—Present, Drs. Hardy, Pennock, and Moore. A ram, about a year old, whose pulse was irregular, but seventy-eight per minute, was selected for the experiment, on account of the slowness of the heart's pulsation, which facilitates the analysis both of the sounds and motion. Sensation was destroyed by blows upon the head, as in the preceding experiment, and the chest opened as before, but the heart beat feebly and irregularly, being congested, and expelling but a small portion of its contents. The sounds were feeble over the right ventricle, (not observed over the left,) and the second soon disappeared entirely; but the first sound remained, whilst the heart contracted, which ceased to beat in a short time.

*Experiment 3d.*—Present, Drs. Hardy, Pennock and Moore. A ram, six months old, was chosen; pulse, 102; respiration, 32. Was struck upon the forehead anterior to the horns. Some difficulty was experienced in introducing the tube connected with the bellows, and in opening the chest. Fifteen minutes elapsed before the heart was exposed. It was found congested and its action irregular. The sounds were more feeble and the heart contracted less forcibly than in the first experiment, but the coincidence between the impulse and ventricular systole were the same, as were also the spiral motion, the peculiar character and succession of the sounds, as well as their comparative intensity at the base and body of the heart. Suspecting from the experiments of others, as well as from the facts we had observed, that the semilunar valves were concerned in the production of the second sound, we attempted to elevate them by hooks introduced into the aorta and pulmonary artery, and note the effect upon the sounds. In consequence of puncturing the artery, hæmorrhage succeeded, and we failed in our purpose. The heart, while still beating, was removed from the body, and the stethoscope applied to the ventricle. It continued to contract many times while in the hand, and during contraction, a sound resembling the first sound was heard, differing only in being more feeble. But one sound was heard. The ventricles were then slit open longitudinally, and emptied of blood, and the same sound was elicited. Pulse fell at one time to 84 per minute. Heart beat three-fourths of an hour.

*Experiment 4th.*—Present, Drs. Hardy, Pennock and Moore. A ram, about a year old, was opened as in experiment 3d. Our attention was now directed exclusively to raising the semilunar valves, but without success. The heart was again removed as in former experiment, the ventricle and right auricle cut open, and emptied of blood, and the fingers thrust into the apertures elevating the tricuspid and semilunar valves. A sound precisely similar to that in the last experiment was detected, but less intense.

*Experiment 5th.*—Present, Drs. Hardy, Wood and Moore. A ram, about a year old. We administered two drachms of Allen's Prussic Acid, containing ten drops of the pure acid. Spasmodic breathing was induced in a few seconds. At the expiration of one minute and a half, the trachea was opened; and respiration established at the end of two minutes. Immediately upon cutting through the integuments, no blood was observed to flow. At the end of four minutes, the heart was exposed, but perfectly motionless and enormously distended.

*Experiment 6th.*—Present, Drs. Hardy, Pennock and Moore, and Mr. Burns. The animal, an ewe, one year old. Deprived of sensation as before. Opened in fifteen minutes. Heart contracted irregularly at first. Same character of first and second sound; same relation of pulse, impulse and ventricular contraction, and same comparative character of sounds upon the left and right ventricles as in first experiment. Heart did not contract vigorously as in first experiment, and when the right ventricle became congested, the second sound disappeared over it. The contractions of the two ventricles were also synchronous. The heart being allowed to rest upon the collapsed lungs, the apex was not observed to rise. The heart during the contraction of the ventricle diminishes transversely, but elongates about one-fourth of an inch, as measured from base to apex. We again failed in elevating the valves. The heart was removed as in experiments 3d and 4th, with the same results.

*Experiment 7th.*—As those experimenters who had preceded us had found greater success upon the calf, we procured one about nine days old. It was deprived of sensation by a blow upon the occiput. Some difficulty was experienced in opening the trachea, and two minutes had elapsed before artificial respiration was commenced; and upon opening the chest, life was extinct; a few very feeble contractions being observed in the right ventricle.

*Experiment 8th.*—A calf, five days old, pulse, one hundred and thirty; respiration, thirty-two. Both sounds heard distinctly through the chest. The animal was struck upon the forehead immediately above the frontal sinus. The chest opened as in first experiment. Same spiral motion observed during contraction. The elongation at the same time one-fourth of an inch, as measured from union of aorta and ventricle to the apex. The whole heart has an apparent motion from the base towards the apex, and the pulmonary artery turns partially around the aorta, which is a fixed point, describing about the arc previously mentioned. The same flattening of right ventricle during its contraction as before observed. When the stethoscope was placed upon the aorta, two inches above the valves, both sounds were heard, but the second sound much louder than the first. Over the pulmonary artery both sounds were faint, but especially the second, which disappeared as the heart became feeble. A curved needle was passed into the aorta, but the sounds were indistinct, and the second appeared to be absent sometimes and not at others when the hook was in the artery. Upon examination after the removal of the heart, it was found that the valves were sometimes elevated, and at others not.

*Experiment 9th.*—Experiencing great difficulty in analysing some of the movements and sounds of the heart in animals of the size upon which we had experimented, we resolved to inspect the heart of a horse, in which the pulse in health ranges from thirty to forty per minute. In this experiment we were assisted by Drs. Gerhard, Stewardson, Peace, Hardy, Fell and Goddard, but to the latter gentleman especially we owe our thanks for the assistance rendered.

We found in the animal we had selected that the pulse was about thirty-six per minute, and respiration twenty-eight in the same time.

In order to prolong life the trachea was opened before the blow was given. Immediately after the blow was struck, which was directed to the forehead, that the skull might be depressed upon the anterior lobes of the brain, the bellows-tube was introduced, and artificial respiration commenced. The skin was dissected back from the median line upon the thorax, the cartilages of the ribs sawn through upon the left side of the sternum, and several of the ribs cut off about one third of their whole length from their sternal extremity. On account of the hæmorrhage, we were obliged to secure many arteries, and twenty-five minutes had elapsed from the time the blow was given until the heart was exposed. It presented the left ventricle, the appendix of the left auricle and a portion of the right ventricle. The pulsations were one hundred per minute, but on account of its size we were enabled to analyse the relative contraction of the auricle and ventricle, which we found to succeed each other as follows:—During the contraction of the ventricle, the auricle dilates; at the expiration of the systole, the auricle contracts with a quick, short motion, and the diastole of the ventricle commences, the auricular contraction apparently occupying about one-half the time of the ventricular diastole. During its systole, the left ventricle flattens and elongates. During its diastole it shortens, and assumes a rounded form. The sounds were detected, but not loud; the second not existing over the pulmonary artery, but heard over the body of the left ventricle.

Death arrested the further progress of the experiment. Dr. Moore coincides with the other gentlemen in reference to the relative contraction of the auricle and ventricle, and thinks his observation in experiment 1st, erroneous.

[Although every experiment had confirmed our views of the agency of the valves of the aorta in the production of the second sound, we had heretofore failed in elevating them; we therefore resolved to pursue the investigation, to ascertain the effect upon the sound, and to exhibit the facts that we had observed to a few medical friends.]

*Experiment 10th.*—Present, Drs. Gerhard, Goddard, Stewardson, Peace, Hardy, Pennock and Moore. A ram, about six months old. Pulse, ninety-six. Deprived of sensation by a blow upon the head, and opened as in experiment 1st. The heart contracted well, but exhibited great irritability when touched. Its pulsations rose to one hundred and fifty per minute, rendering it difficult to analyze the sounds; but the first sound and impulse were observed to coincide. The spiral motion and elongation were as heretofore detailed. While still contracting forcibly, the heart was removed from the body, and the first sound heard when entire, and also when both ventricles were cut open and emptied of blood.

*Experiment 11th.*—As the last experiment had not been very satisfactory, the same gentlemen being present, we pursued the investigation upon a calf, four weeks old. Pulse, one hundred and five. Both sounds distinctly heard through the chest. Struck upon anterior portion of the cranium, and opened as before. The pericardium was left entire, to avoid the irritation of immediate contact with the heart. The stethoscope was placed alternately upon the aorta, the body of the right ventricle, and upon the septum, near the apex. Upon the aorta the second sound was found to predominate; upon the body of the right ventricle it was scarcely heard, and the first was present; and near the apex upon left ventricle or septum both were detected, the first louder. The spiral motion, the elongation and elevation of the apex as before observed. A hook was passed into the aorta by Dr. Moore, and one of the semi-lunar valves elevated; the eyes of the auscultator were closed, to prevent the possibility of bias from preconceived opinions. While in this position the auscultator announced the absence of the second sound, and the accession of a rough bellows sound in the first sound. The hook was then withdrawn, and the second sound was declared to have returned. This experiment was tried twice by each, and by some three times in succession, and the results were uniform. No hook was passed into the pulmonary artery, inasmuch as no sound was heard over it at this time. The auricle contracted while in the hand, emptied of blood.

*Experiment 12th.*—A ram, six months old. Present, Drs. Stillé, Hardy, Pennock and Moore. Pulse, ninety-six; respiration, fifty-six. Animal struck upon forehead, as in previous experiments, and artificial respiration established in three-fourths of a minute. During the opening of the chest, much hæmorrhage took place. The heart was at first tumultuous in its action, but became regular in a few minutes. The first and second sound were heard over the body of the right ventricle, but more feebly than over the left; both sounds were heard over the left ventricle and aorta, but the second louder than the first over the latter than over the former. Hooks were passed into the ventricle, for the purpose of keeping open the auriculo-ventricular valves. (These, however, failed of effecting the object, as seen upon examination afterwards.) The sounds gradually became more feeble, as the heart congested and the second sound ceased altogether, both over the heart and arteries, while the first still remained. The auricle was observed to contract over its entire surface, as much upon the body as upon the appendix The contractions with reference to the ventricles were irregular at this time, except for a very short period, when they appeared to precede those of the ventricle immediately, recurring at the termination of repose. The heart contracted one hour after the blow was given.

*Experiment 13th.*—Wether, nine months old. This experiment failed on account of defect in the apparatus. As the heart became more feeble the auricle appeared to contract immediately antecedent to the systole of the ventricle, but owing to the circumstances attendant upon this experiment, we feel very uncertain as regards the observation.

*Experiment 14th.*—Ewe, nine months old. Struck as before. Trachea opened in half a minute. Chest opened in four minutes. Heart tumultuous. It gradually became more quiet, until it fell to one hundred and twenty, and contracted forcibly. The first sound alone was heard over the right ventricle and pulmonary artery. Pressure upon this artery produced a bellows sound in the first sound. The auricles were pushed into the auriculo-ventricular openings by the fingers. The first sound was thus rendered much more feeble, and lost its sharp character; the ventricles contracting imperfectly and irregularly.

*Experiment 15th.*—A calf, five days old, pulse, one hundred and twenty-six; respiration, thirty. Destroyed by a blow upon the head, as before. Artificial respiration established in two minutes and a half. The heart was exposed in six minutes, rather hurried in its action, but soon fell to one hundred and twenty pulsations per minute. The heart contracted with a moderate force. The second sound extremely feeble over the body of the right ventricle and pulmonary artery; but it soon disappeared over both. The sound was still heard over the left ventricle and aorta, louder over the latter. The auricle contracted with a quick motion, the contraction not being confined to the appendix, but extending over the whole body of the organ. As the heart became weaker, the pulsations were slower, and we were enabled to analyze the relative contractions of the auricle and ventricle much better than at any previous experiment. They evidently bore a different relation from what we had previously supposed. The succession is as follows:—First the auricle contracts, and the action is immediately propagated to the ventricle, which contracts instantly, accompanied with the diastole of the auricle; the diastole of the ventricle immediately follows, accompanied with a subsidence of the auricle by passive and not active contraction, which partially fills the ventricle; then follows the state of repose, at the termination of which, the auricle contracts. During the dilatation of the auricle, the vena cava also dilates, but it was difficult to say whether the cava dilated during the contraction of the auricle or not, as the contraction of the latter was so rapid and so soon followed by the contraction of the ventricle. While still contracting, and when scarcely any sound was heard upon the ventricles, the stethoscope was applied to each auricle, and a sound similar to the first was heard, but very short, and more flapping, resembling very nearly the first sound of the fœtal heart.

*Experiment 16th.*—A calf, two months old. Pulse ninety. Deprived of sensation as before. The chest was opened in eight minutes, and a few ribs removed from the left side. The heart pulsated slowly, and at a rate of ninety-five per minute; both sounds were distinct, but not loud. The second sound was heard more loudly over the pulmonary artery than on the right ventricle, the sound being but feeble in either position. Both sounds were heard upon the left ventricle. An instrument was introduced into the left ventricle, through the auricle, and the mitral valves prevented from collapsing; this produced congestion of the ventricle immediately, and the action became hurried and irregular. The stethoscope being applied to the left ventricle, the sound was not as loud and clear as before, but not modified in any other manner. The instrument was then withdrawn, and the sound became louder. The relative contractions of the auricles and ventricles were as in the last experiment.

The difference in the intensity of the first sound in this experiment, when the mitral valve was kept open and when allowed to close, may be attributed to the fact that there was no fixed point for the muscle of the ventricle to act upon, by the retention of the blood, and it therefore could not empty itself of its contents, and, of course, would not yield a strong sound.

From these experiments we draw the following conclusions:

1st. The impulse is synchronous with, and caused by the ventricular contraction,—and when felt externally, arises from the striking of the apex of the heart against the thorax.

2d. The expulsion of the blood from the ventricles is effected by an approximation of the sides of the heart only, and not by a contraction of the apex towards the base; during the systole the heart performs a spiral movement, and becomes elongated. (Experiments 6th, 10th, and 11th.)

3d. The ventricle contracts and the auricle dilates at the same time, occupying about one-half of the whole time required for contraction, diastole, and repose. Immediately at the termination of the systole of the ventricle, its diastole succeeds, occupying about one-fourth of the whole time, synchronous with which the auricle diminishes, by emptying a portion of its blood in the ventricle, unaccompanied with muscular contraction. The remaining fourth is devoted to the repose of the ventricles, near the termination of which the auricle contracts actively, with a short, quick motion, thus distending the ventricles with an additional quantity of blood: this motion is propagated immediately to the ventricles, and their systole takes place, rendering their contractions almost continuous. (Exp. 15 and 16.)

4th. From the termination of their diastole to the commencement of their systole, the ventricles are in a state of perfect repose, their cavities remaining full, but not distended, while those of the auricles are partially so, during the whole time.

5th. The sounds are produced by the motions of the heart or its contents, and not by striking against the thorax, as proved in all the experiments; being much louder when the stethoscope was applied directly to the heart, than when to the chest, or with the lungs interposed.

6th. The sounds are more distinct when the muscle is thin, and contracts quickly. Hence, the clear, flapping character of the first sound over the right ventricle, as compared with the left.

7th. The first sound, the impulse, and the ventricular systole, are synchronous. This sound may be a combination of that caused by the contraction of the auricles, the flapping of the auriculo-ventricular valves, the rush of blood from the ventricles, and the sound of muscular contraction. From experiments 3d, 4th, 6th, and 10th, when the heart was removed from the body, the ventricles cut open and emptied of their contents, the auriculo-ventricular valves elevated, and a sound, resembling the first, still heard, it may be chiefly attributed to the muscular contraction. That these valves aid but slightly in its production, may also be inferred from experiment 16.

8th. The second sound is caused exclusively by the closure of the semi-lunar valves, from the tendency to regurgitate of the arterial column of blood upon them. This is proved by the greater intensity of this sound over the aorta than elsewhere, the blood having a strong tendency to return through the valvular opening; by the greater feebleness of the sound over the pulmonary artery, which is short, and soon distributes its blood through the lungs, thus producing but slight impulse upon the valves in the attempt to regurgitate; by the disappearance of the sound, when the heart becomes congested and contracts feebly; and, finally, on account of its entire extinction when the valve of the aorta was elevated.

9th. The second sound is synchronous with the diastole of the ventricle.

From these experiments, it will be seen that our conclusions coincide very nearly with those of the British physiologists,—the correctness of whose results, when compared with those of the French, may be mainly attributed to the use of larger animals. From our observations, calves, of from four to eight weeks old, are decidedly preferable to other quadrupeds for these investigations. The tenacity of life of calves of this age is greater than in older animals, whilst the cardiac pulsations are slower, and more forcible, than they are in the younger. The heart of this animal, too, is of large size, and the introduction of hooks for the elevation of the valves is readily effected.

[The English and Irish physiologists enjoyed great facilities in the slow and regular action of the heart, as induced by the woorara. Perhaps, at some future period, when this may be obtained, the investigations may be pursued, as other points of inquiry are offered.]

(Signed) C. W. Pennock,
M. Moore.

*Philadelphia*, *Nov.* 2, 1839.

